# Performance of *Sesamia nonagrioides* on cultivated and wild host plants: Implications for Bt maize resistance management

**DOI:** 10.1002/ps.5913

**Published:** 2020-06-13

**Authors:** Ana M Camargo, María Arias‐Martín, Pedro Castañera, Gema P Farinós

**Affiliations:** ^1^ Dept. of Microbial & Plant Biotechnology centre es Centro de Investigaciones Biológicas Margarita Salas Madrid Spain

**Keywords:** Mediterranean corn borer, alternative hosts, resistance management, MON 810, HDR strategy, unstructured refuges

## Abstract

**BACKGROUND:**

*Sesamia nonagrioides* is an important maize pest in the Mediterranean basin that is effectively controlled by Cry1Ab‐expressing maize (Bt maize). The continued cultivation of Bt maize in Spain exerts high selection pressure on the target pests, which could lead to the development of resistance. Provision of refuges of non‐Bt plants is an essential component in the high‐dose/refuge (HDR) strategy to delay resistance evolution. Here we analyze the suitability of cultivated (rice and sorghum) and wild (Johnsongrass, cattail, common reed and giant reed) plants, reported as hosts of *S. nonagrioides*, for larval development and oviposition of this pest compared to maize, and we evaluate their potential role in delaying resistance development to Bt maize.

**RESULTS:**

Bioassays conducted with plant pieces or whole plants showed that the larval cycle could only be completed in the three cultivated plants and in Johnsongrass. Females showed a strong preference for ovipositing on maize in comparison with sorghum or rice. Although young larvae consumed more sorghum than maize in two‐choice bioassays, both larvae and adults had a better performance (shorter larval period and higher pupal weight, fecundity and fertility) when larvae fed on maize throughout their larval stage than when they fed on sorghum or rice.

**CONCLUSION:**

None of the alternative hosts of *S. nonagrioides* tested here should be considered as natural unstructured refuges within the HDR strategy for Bt maize and this pest in Spain, as some of the necessary requirements to fulfill this strategy would not be met. © 2020 The Authors. *Pest Management Science* published by John Wiley & Sons Ltd on behalf of Society of Chemical Industry.

## Graphical abstract

*Sesamia nonagrioides* performance on alternative hosts is worse than on maize, its main host. Thus, they do not meet the necessary requirements to serve as unstructured refuges for Bt maize.

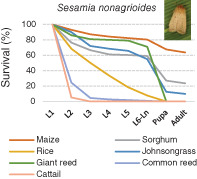

## 1 INTRODUCTION

The Mediterranean corn borer, *Sesamia nonagrioides* Lefèbvre (Lepidoptera: Noctuidae), is the most damaging pest of maize in the Mediterranean basin.^1^ First instar larvae of this species bore into maize plants shortly after the eggs hatch and feed inside the stalks for the rest of the larval stage,^2^ which greatly limits the efficacy of chemical sprays to control them.^3^ The introduction in Spain of genetically modified (GM) maize varieties that express the *Bacillus thuringiensis* toxin Cry1Ab (Bt maize) in the late 1990s marked a breakthrough in the management of this pest, given their high effectiveness in its control. This led to the rapid implementation of this technology in Spain, especially in areas of high infestation like the Ebro Valley (northeast of Spain), where yield losses were greatest,^4^, ^5^ so that about 60% of all the maize grown in this area in the last 5 years is Bt.^6^, ^7^

The intensive and continuous cultivation of Bt maize exerts a high selective pressure over the pests they target and could lead to resistance evolution, considered as one of the main threats for its long‐term sustainability.^8^, ^9^ The strategy known as high‐dose/refuge (HDR), adopted in the European Union (EU) to delay resistance evolution to Bt crops, involves (i) using crop varieties that produce high concentrations of the Bt toxin, capable of killing all or nearly all individuals heterozygous for resistance,^10^ and (ii) setting refuges close to the Bt fields. In principle, refuges can be non‐Bt varieties of the Bt crop or any other plant species, as long as they produce large enough pest populations consisting of viable, susceptible insects that could mate with the potentially resistant homozygous individuals that might survive in the Bt crop.^11^, ^12^, ^13^ Structured refuges, i.e. allotting an area near to the Bt field to growing varieties of the same crop not expressing the Bt trait, are the most commonly used type of refuge. For instance, their adoption is mandatory in the EU, where they have proved to successfully delay resistance development to Bt maize in the target pests *S. nonagrioides* and *Ostrinia nubilalis* Hübner,^14^, ^15^ and also in the USA for the cultivation of Bt maize expressing one insecticidal protein and targeting Lepidopteran pests.^16^ Unstructured refuges, on the other hand, consider alternative host plants of the target pest, such as other crops and weeds that grow close to the Bt crop, as the source of susceptible individuals.^17^ These unstructured refuges have been shown to be effective in delaying resistance evolution to Bt cotton of different polyphagous pests in China and the USA,^18^, ^19^ and their use is approved as part of the resistance management program of pyramided Bt cotton in some areas of the USA.^16^ Moreover, some authors have argued that in some cases using natural refuges could reduce and sometimes even suppress the need to plant structured refuges composed of non‐Bt varieties.^20^ The potential of alternative plant species used by *S. nonagrioides* to serve as unstructured refuges in Bt maize resistance management has not been studied to the date. To evaluate whether alternative hosts could be useful in insect resistance management (IRM) plans it is key to evaluate the performance of this noctuid pest on these plant species, as well as its preference between them and its main host, maize, to learn whether large and healthy populations of the pest could build up in the alternative hosts. In this context, determining the free amino acid and soluble sugars content in the tested plant species could provide important information, since these parameters have been reported to influence oviposition preference and to stimulate larval feeding in a range of insect species, including noctuids.^21^, ^22^

The inadequate or poor implementation of IRM strategies to delay resistance evolution has led several insect pests to develop resistance to a range of Bt crops expressing different Bt toxins.^23^ *Sesamia nonagrioides*, however, has remained susceptible to Cry1Ab expressing maize varieties, which have been cultivated in Spain for over 15 years,^15^ even though a resistance allele to Bt maize has been reported in a population from the Ebro Valley.^24^ A resistance evolution model for *S. nonagrioides* was recently developed considering more than 20 variables known to affect the rate of resistance development, including aspects of the pest biology and genetics as well as agronomic practices in that area.^4^ This model considered *S. nonagrioides* as a functional monophagous species on maize, its primary host, in the Ebro Valley. Nevertheless, despite its high level of specialization in maize, *S. nonagrioides* has shown a certain degree of polyphagy, since it has been recorded in a wide range of cultivated and wild host species of the Poaceae, Cyperaceae and Thyphaceae families across its distribution range.^25^, ^26^ Regarding cultivated plants, *S. nonagrioides* is known to be a major pest of sorghum^27^, ^28^ and rice.^29^, ^30^ The degree of polyphagy has usually been considered an important factor for resistance development, although some studies have not found a clear causal relationship between both evolutionary phenomena.^31^ In the case of Bt crops, resistance evolution is expected to occur faster in monophagous pests that feed on the modified plant species, which are subjected to a higher selective pressure in comparison with polyphagous pests.^32^, ^33^

Gaining knowledge about the range of hosts that can be used by *S. nonagrioides* and its preference for them is essential for the management of this corn borer in Bt maize. Furthermore, the use of different types of refuges (crop *vs* non‐crop) is also important in the context of IRM strategies, as changes in agronomic practices affecting the availability of these hosts may influence the effectiveness of IRM programs.^34^ Thus, the aim of this study was to investigate whether a range of cultivated and wild plants, reported to be potential hosts of *S. nonagrioides*, are suitable for the larval development and oviposition of this pest, as compared to maize, its main host. The results obtained will shed light on the possibility of considering these plants as unstructured refuges for Bt maize within the HDR strategy, which in turn will help to improve the management of resistance of *S. nonagrioides* to this GM crop and contribute to fine‐tune the *S. nonagrioides* resistance evolution model.

## 2 MATERIALS AND METHODS

### 2.1 Plant material

We tested the suitability for oviposition and larval performance of *S. nonagrioides* of three cultivated plants [*Zea mays* (maize), *Oryza sativa* (rice) and *Sorghum bicolor* (sorghum)] and four wild host plants that are frequently found within maize fields [the weed *Sorghum halepense* (Johnsongrass)] or close to them [*Typha domingensis* (cattail), *Phragmites australis* (common reed) and *Arundo donax* (giant reed)]. All species belong to the family Poaceae, except cattail, which belongs to the family Typhaceae.

Maize (var. DKC4795) and sorghum (var. Express Rojo) plants were grown in potting soil (Compo Sana Universal, CompoAgricultura S.L., Barcelona, Spain), whereas rice plants (var. Gleva) were grown on a mixture of 63.5% peat, 36.5% vermiculite and 0.63 g of CaCO_3_ per liter of soil. Johnsongrass (collected in San Fernando de Henares, Madrid, Spain) and giant reed (Instituto Nacional de Tecnología Agraria y Alimentaria, Madrid, Spain) were also grown in potting soil, whereas a mixture of 50% potting soil and 50% river sand was used to grow cattail (collected in the stream Pantueña, Madrid, Spain) and common reed (Ecodena S.L., Sevilla, Spain). All plants were grown in 25 cm diameter × 24 cm high pots, and maintained in a greenhouse at 25 ± 3 °C, relative humidity of 75 ± 10% and 16:8 (L:D) photoperiod.

### 2.2 Insect rearing

Insects used in the assays came from a laboratory colony of *S. nonagrioides* of the Centro de Investigaciones Biológicas (Madrid, Spain) reared on a meridic diet, as described in González‐Núñez *et al*.^3^ The oviposition cages consisted of a 12.5 cm diameter × 12 cm high pot with 8–10 V3 maize seedlings, enclosed by a 12 cm diameter × 30 cm high see‐through and colorless Plexiglas cylinder covered on top by a mesh that allowed ventilation, and 8–10 pairs of adults were placed inside. After 7 days, egg clusters were collected from the plants and placed on moistened filter paper for egg hatching. The whole rearing process took place in growth chambers (Sanyo MLR‐350 H, Sanyo, Japan) at 25 ± 0.3 °C and 16:8 (L:D) photoperiod.

### 2.3 Performance of *S. nonagrioides* on different host plants

#### 2.3.1 *Preliminary oviposition bioassay*

A preliminary no‐choice oviposition test using 10–20 replicates per plant species, each of them consisting of three confined pairs of *S. nonagrioides* per arena, was performed to confirm that the seven hosts selected according to the available bibliography were suitable for oviposition in the conditions used for this study. The results showed that females laid a significant number of fertile eggs in all the plants (on average, more than 400 eggs were recovered per replicate, data not shown). Therefore, larval performance was assessed on all seven hosts in two ways: by using excised parts of leaves and stems and using whole plants.

#### 2.3.2 *Performance on excised parts of plants*

Individualized neonates (<24 h) were confined in plastic boxes 4 cm diameter × 2 cm high and fed *ad libitum* with fresh pieces of leaves and stems of each plant species. All boxes were examined daily, and the dates of molting, pupation and adult emergence were recorded. Between 60 and 144 larvae were used for each plant species. A control to ensure that the population was in optimal condition was set up with 102 larvae fed with the same meridic diet used to rear the laboratory population. The length of the larval cycle was determined by counting the number of days it took each larva to reach the pupal stage from the start of the experiment, and the longevity of the adults was calculated as the number of days between emergence and death of the adult. Pupae were weighed 24 h after pupation. To evaluate dietary effects on adult performance, individual pairs of adults were placed in arenas consisting of 6 cm diameter × 6 cm high pots with three V3 maize seedlings confined by a ventilated, see‐through plastic cylinder (5.4 cm diameter × 15.5 cm high) for mating and oviposition. Egg clusters were collected 7 days later and the number of eggs determined using a stereomicroscope (Leica M125, Leica Mycrosystems, Germany). Eggs were placed on top of moistened filter paper in plastic boxes for hatching and their viability recorded. These assays were carried out in growth chambers at 25 ± 0.3 °C and 16:8 (L:D) photoperiod. Additionally, the standardized growth index (SGI) was estimated for each larva of each host species tested^35^:

SGI = pupal weight (mg)/length of larval period (days).

#### 2.3.3 *Performance on whole plants*

Six neonates (<24 h) of *S. nonagrioides* were placed on the leaf sheaths of leaves 3, 4 and 5 (two larvae per leaf), with the exception of *P. australis*, in which three neonates were used (one per leaf) due to the narrow diameter of the stem in this species. The main stem of rice plants was considered for infestation. Plants were then confined within a ventilated methacrylate cylinder and watered regularly during the running time of the experiment. Between 25 and 27 days after infestation the plants were dissected and the larval recovery rate, measured as the percentage of initial larvae that were recovered at the end of the bioassay, was recorded in each plant, as well as larval weight and larval stage (L1–L6 for first to sixth instars, respectively) of the recovered larvae. The assays were performed with V6–V8 plants of all plant species except rice, which was used when the plants reached the panicle formation phase, and they took place in a greenhouse, using 7–22 plants per species, at 25 ± 3 °C and 16:8 (L:D) photoperiod.

### 2.4 Larval feeding preference

Feeding preference was evaluated by two‐choice and no‐choice bioassays. Since maize is the primary host of *S. nonagrioides* in Spain, this species was used as the reference host for comparison with the other two cultivated hosts, rice and sorghum. The three species were planted at the same time and offered to *S. nonagrioides* females when maize plants reached the V8 phenological stage. These experiments were performed using leaf disks as an appropriate proxy to assess feeding preferences of *S. nonagrioides* larvae.

#### 2.4.1 *Two‐choice assays*

Two‐choice assays were conducted to examine feeding preferences of *S. nonagrioides* larvae between maize and rice or sorghum. Given that this corn borer is known to feed on the three tested species, feeding preference was considered as a significantly higher consumption of one of the two species used in the assay. The choice arena consisted of a Petri dish (60 mm diameter × 5 mm high) coated on its bottom with a 2.5% agar solution. Leaf disks (8 mm diameter) containing the mid‐rib were excised from rice, maize and sorghum plants with a cork borer and fitted into the holes punched in the agar layer, alternating leaf disks of the two species (maize‐rice or maize‐sorghum) in the agar arena. A recently molted (<24 h) second instar larva weighing 0.50–1.25 mg (mean ± 1SD) was placed in the center of the dish after a 6‐h starvation period. All dishes were sealed and placed in a growth chamber at 25 ± 0.3 °C and complete darkness for the duration of the assay. Twenty replicates of each combination were evaluated. The experiment concluded when larvae in an external control that only contained maize disks had consumed approximately 50% of the plant material. Both the initial and final fresh weights of larvae and leaf disks, measured separately for each plant species, were recorded. Larvae were then frozen at −20 °C and afterwards dried in an oven at 60 °C for 48 h to estimate their dry weight. Uneaten leaf disks were cleaned of frass and oven‐dried following the same procedure.

The preference index proposed by Kogan and Goeden (1970)^36^ was calculated as a measure of larval preference on a dry weight (DW) basis:

preference index (*C*) = 2*A*/(*M* + *A*) where *A* is the consumption of alternative host (%DW) and *M* is the consumption of primary host (%DW). This index can range between 0 and 2, so that *C* = 1 indicates that larvae do not feed preferentially on either plant, whereas values lower than 1 denote a preference for the primary host and values higher than 1 indicate larvae feed preferentially on the alternative host.

Additionally, the nutritional indices described by Farrar *et al*.^37^ were calculated. The relative consumption rate (RCR) was estimated separately for the two plant species in each two‐choice assay:

*RCR* = *DW*_*i*_ – *DW*_*f*_)/*LW*_*i*_ × *D* where *DW*_i_ is the initial dry weight of leaf disks (mg), *DW*_f_ is the final dry weight of leaf disks (mg), *LW*_i_ is the initial larval dry weight (mg) and *D* is the duration of the assay (days). Initial dry weight of leaf disks was calculated from their fresh weight using an equation that relates both parameters, obtained for each plant species by weighing 10 batches of six freshly excised leaf disks and weighing them again after 48 h at 60 °C. Similarly, *LW*_i_ was calculated using an equation obtained by measuring the fresh and dry weights of 373 L2 larvae in the same weight range as those used in the assays. All weights were determined using an analytical balance (Mettler‐Toledo AX205, Mettler‐Toledo International Inc., Columbus, OH, USA).

#### 2.4.2 *No‐choice assays*

These tests were performed similarly to two‐choice assays, but all disks in the agar arena corresponded to the same plant species. Seventeen replicates were tested for maize, 16 for sorghum and eight for rice. In this case, the experiment concluded when larvae in the maize assay had consumed approximately 75% of leaf disks. Three nutritional indexes were estimated: the RCR (described above), the relative growth rate (RGR) and the efficiency of conversion index (ECI),^37^ so that:

*RGR* = (*LW*_f_ – *LW*_i_)/*LW*_i_ × *D* where *LW*_f_ is the final larval dry weight (mg), *LW*_i_ is the initial larval dry weight (mg) and *D* is the duration of the assay (days), and

*ECI* (%) = (*RGR*/*RCR*) × 100.

### 2.5 Oviposition preference on cultivated hosts

Two‐choice assays were carried out to determine the oviposition preference of females between the primary cultivated host (maize) and rice or sorghum. For each replicate, two males and a female were confined in a choice arena consisting of a 25 cm diameter × 24 cm high pot with one maize plant and either a rice or a sorghum plant, covered with a mosquito net with a diameter of 56 cm and a height of 230 cm, attached by a cable to the roof of the greenhouse, that gave the moths enough space to move freely. All host species were sown at the same time and exposed to *S. nonagrioides* adults when maize plants reached the V8 phenological stage. After 7 days, all the adults were recovered and plants were examined. Fecundity was estimated as the total number of eggs laid per female and plant and egg viability was estimated a week later as described in section 2.3. Female moths were dissected to check their mating status, and only replicates in which a mated female was recovered were considered as valid. Twenty replicates of each option (maize‐rice or maize‐sorghum) were set up. These assays took place in the greenhouse at 25 ± 3 °C and 16:8 (L:D) photoperiod.

### 2.6 Free amino acid and free sugar content of the cultivated hosts

The free amino acid and free sugar content was assessed in maize, sorghum and rice leaves used in the feeding assays to determine if they could have an effect on the choice of the plants, since these compounds have been proved to influence insect preference and performance between hosts in some lepidopteran species.^38^, ^39^

The extraction method followed to estimate the quantity of free amino acids was that used in Ximenez‐Embún *et al*.,^40^ based in the technique described in Hacham *et al*.^41^ Three samples (20–30 leaf disks/sample) of each plant species were frozen at −80 °C and grinded using a mortar to obtain approximately 100 mg of leaf material per sample, whereupon 600 μL of water:chloroform:methanol (3:5:12 v/v/v) extraction buffer was added to each sample. This was followed by a 4‐min centrifugation at 4 °C and 14 000 rpm, after which the supernatant was transferred to a new tube and the pellet was resuspended in 600 μL of extraction buffer and centrifuged again. The supernatant was pooled with that obtained in the previous centrifugation and 300 μL of chloroform and 450 μL of double‐distilled water were added to each sample. After a final 2‐min centrifugation of the samples at the same temperature and speed, the top layer of the solution containing the amino acids was transferred to a new tube and placed in a SpeedVac Concentrator Savant SVC‐100H (ThermoFisher Scientific, Wilmington, DE, USA) overnight. When all the solvent was evaporated, the samples were taken to the Protein Chemistry Service at the CIB (CSIC, Madrid), where their amino acid content was determined using a Biochrom 30 Amino Acid Analyser (Biochrom, Cambridge, UK). For this purpose, the samples were first resuspended in 100 μL of sodium citrate loading buffer at pH 2.2 and 10 μL of each sample was injected in the analyzer. The free amino acid content in each sample was estimated on a dry weight basis.

Determination of the plants' free sugar content was performed on dry plant material. Leaf disks excised from the leaves used in the assays were dried at 75 °C for 48 h and then ground to obtain a fine powder. Three samples (≈3 mg of leaf powder per sample) were considered per plant species. Each sample was homogenized in 650 μL of 95% ethanol and heated at 80 °C for 20 min, followed by centrifugation at 10000 rpm for 10 min and collection of the supernatant in a tube. This process was repeated two more times. The supernatants of each sample were pooled and divided into two 750 μL replicates, which were dried in a SpeedVac Concentrator Savant SVC‐100H for approximately 12 h. Each sample was then resuspended in 500 μL of double‐distilled water and 1 mL of 0.2% anthrone in 95% sulfuric acid (v/v) was added to each of them. After 15 min of incubation at 90 °C the absorbance of each sample at 630 nm was measured in a VERSAmax microplate reader (Molecular Devices Corp., Sunnyvale,USA).

### 2.7 Statistical analysis

Prior to the statistical analysis of the results, normality (Kolmogorov–Smirnov test) and homocedasticity (Levene test) were checked in all variables, and those that did not comply with these requirements were transformed to arcsin√*x* or log(*x* + 1) for percentages or continuous variables, respectively. A significance level of α = 0.05 was considered, and all analyses were performed using the statistical software SPSS (SPSS Statistics 24.0, IBM, USA).

In larval development assays using parts of plants, one‐way ANOVA followed by either a Dunnett's *t*‐test (when variances were homogenous) or a Dunnett's T3 test (when variances were not homogenous)^42^ were carried out to study whether length of the larval cycle, pupal weight, adult longevity and SGI in the different plant species differed significantly from the values recorded in maize. A Student's *t*‐test was carried out to check for differences between host species in fecundity and fertility for adult pairs resulting from larvae fed on maize and sorghum, the only two species in which adults could be set up for mating and oviposition. In assays that considered whole plants, one‐way ANOVA followed by Dunnett's tests were used as described above to study whether larval recovery rate and mean weight per instar were different in the alternative hosts in comparison with maize.

Differences in fecundity and fertility between maize and sorghum or rice were analyzed by paired Student's *t*‐tests, comparing the values resulting from subtracting the value of the variable measured in the alternative host from the value measured in maize. Likewise, differences in RCR between species in larval feeding two‐choice assays were analyzed with paired Student's *t*‐tests following the same procedure.

In no‐choice larval feeding assays, one‐way ANOVA followed by a Dunnett's *t*‐ or T3 tests was performed to determine if host species had a significant effect on RCR, RGR and ECI. Differences between alternative hosts and maize in their free sugar and total free amino acid content were analyzed with one‐way ANOVA followed by Dunnet's *t*‐tests.

## 3 RESULTS

### 3.1 Performance of *S. nonagrioides* on different host plants

#### 3.1.1 *Performance on excised parts of plants*

**Figure 1:**
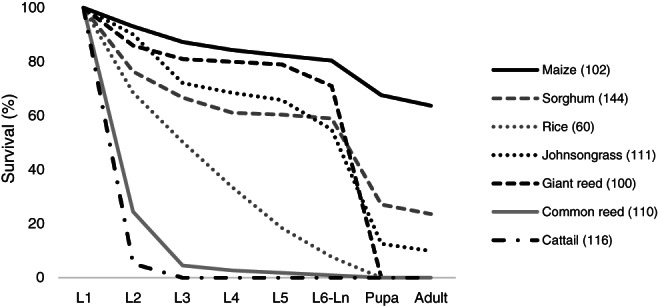
Survival rates of the different stages of *S. nonagrioides* fed on pieces of leaf and stem of seven host plants. Number of larvae tested per plant species is shown in brackets.

*Sesamia nonagrioides* only reached the adult stage when larvae were fed with pieces of three out of the seven tested plant species: maize, sorghum and Johnsongrass, with survival rates to the adult stage of 63.7%, 23.6% and 9.9%, respectively. Larvae fed on common reed and cattail died mostly during the early larval stages, while larvae fed on rice and giant reed died at more advanced larval stages, so that no pupae were obtained in any of these four species (Fig. 1). Larvae feeding on maize usually underwent five molts before they completed their larval stage, whereas supernumerary molts were common in individuals fed on the alternative hosts. A high survival rate to adulthood was recorded in larvae fed on a meridic diet (91.2%), indicating the good health of the *S. nonagrioides* population used in the experiments.

Focusing on the three plant species where *S. nonagrioides* completed its development (maize, sorghum and Johnsongrass), it had a better performance when fed with maize than with any one of the others, as indicated by the significantly shorter duration of the larval development and the higher adult longevity, SGI and pupal weight observed in larvae fed on maize (Table 1). Average fecundity and fertility were also higher in adult pairs derived from larvae fed with maize. Since females and males emerged at different times, no couples could be set up for mating and oviposition in adults resulting from larvae fed with Johnsongrass (Table 1).

**Table 1:** Performance of *S. nonagrioides* fed on plant parts (leaves and stems) of different plant species (mean ± SE)

Host	Larval development (days)	Pupal weight (mg)	SGI^¶^ (mg day^−1^)	Adult longevity (days)	Fecundity	Fertility (%)
Maize^a^	30.9 ± 0.4	188 ± 5	4.6 ± 0.1	8.7 ± 0.3	539 ± 32	88.2 ± 1.8
Sorghum^b^	48.3 ± 1.2*	128 ± 3*	2.7 ± 0.1*	5.1 ± 0.2*	125 ± 31	42.1 ± 14.0
Johnsongrass^c^	46.1 ± 1.7*	113 ± 5*	2.0 ± 0.1*	5.1 ± 0.5*	–	–
*F / t* (p)	162.22 (<0.001)	56.19 (<0.001)	66.89 (<0.001)	10.40 (<0.001)	108.13 (<0.001)	3.04 (0.011)

Only those species in which *S. nonagrioides* completed its life cycle are shown.

^*^ Significant differences from values obtained in maize (one‐way ANOVA followed by Dunnett's *t*‐test, *P* < 0.05).

^a^ Sixty‐five adults emerged from larvae fed on maize; 16 couples were set up for mating and oviposition.

^b^ Thirty‐four adults emerged from larvae fed on sorghum; 10 couples were set up for mating and oviposition.

^c^ Eleven adults emerged from larvae fed on Johnsongrass. Female and male adults emerged at different times, so no couples could be set up.

^¶^ SGI, standardized growth index.

#### 3.1.2 *Performance on whole plants*

**Figure 2:**
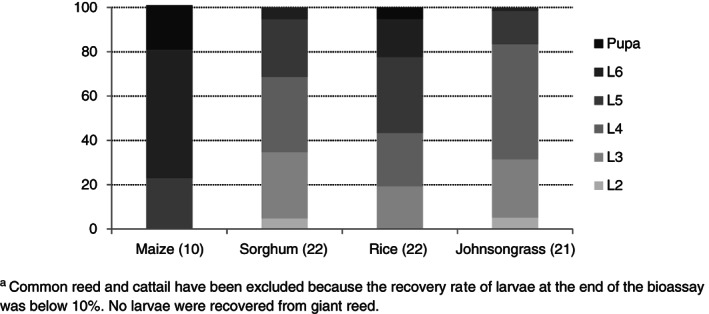
Frequency (%) of the different stages of the life cycle in *S. nonagrioides* recovered from infested plants. Common reed and cattail have been excluded because the recovery rate of larvae at the end of the bioassay was below 10%. No larvae were recovered from giant reed. Number of replicates per plant species is shown in brackets.

**Figure 3:**
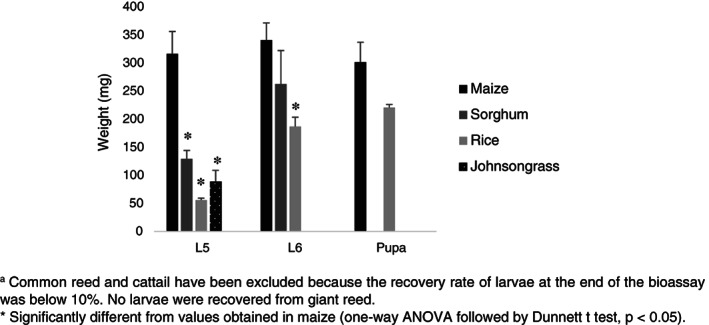
Larval and pupal weight per host species a (mean ± SE) in *S. nonagrioides* recovered from infested plants. Common reed and cattail have been excluded because the recovery rate of larvae at the end of the bioassay was below 10%. No larvae were recovered from giant reed. *Significantly different from values obtained in maize (one‐way ANOVA followed by Dunnett's *t*‐test, *P* < 0.05).

Individuals of *S. nonagrioides* were recovered 25–27 days post infestation in six out of the seven plant species tested, whereas no larvae were recovered from giant reed. The recovery rate of larvae at the end of the bioassay was very low in common reed and cattail (5.6% and 5.0%, respectively), so these plants were excluded from the statistical analyses. When the experiment was stopped, nearly 4 weeks post infestation, all larvae were expected to be at least L5, since the average duration of the larval stage in *S. nonagrioides* larvae reared at 25 °C and a 16:8 photoperiod was estimated in 32.5 days.^43^ However, only in maize all the larvae recovered were L5 or bigger (22.5% L5, 57.5% L6 and 20.0% pupae). Nearly 69% of the larvae recovered from sorghum were L2–L4, and around 31% were L5–L6 larvae. Likewise, 83.1% of the larvae recovered from Johnsongrass were L2–L4 larvae, and 16.9% were L5–L6 larvae. Finally, 42.5% of the individuals recovered from rice plants were L3–L4 larvae, and 57.5% were L5–pupae (Fig. 2). Average larval weight was significantly higher in L5 larvae recovered from maize than in those recovered from sorghum, rice and Johnsongrass. Sixth instar larvae and pupae were also heavier when recovered from maize plants with regard to those from rice plants, whereas the difference was not significant in the case of L6 larvae recovered from sorghum (*P* = 0.054) (Fig. 3).

### 3.2 Larval feeding preference

#### 3.2.1 *Two‐choice assays*

When larvae of *S. nonagrioides* could choose between maize and rice or sorghum, they exhibited different feeding preferences. Larvae chose to feed on maize rather than on rice when they were provided with both species, as shown by the significantly higher value of the relative consumption rate in maize (*t* = 3.78, *P* = 0.001). However, this index had a significantly higher value in sorghum when larvae could choose between this species and maize (*t* = −2.33, *P* = 0.031) (Table 2). These results are supported by the values of the feeding preference index (*C*), which indicated that *S. nonagrioides* larvae preferred maize to rice in rice‐maize assays (0.80 ± 0.09), whereas sorghum was the preferred foliar tissue in maize‐sorghum (1.20 ± 0.10) assays.

**Table 2:** Relative consumption rate (mean ± SE) of *S. nonagrioides* on the three cultivated plant species using two‐choice assays

Choice assay (*n*)	RCR maize (mg mg^−1^ day^−1^)	RCR rice (mg mg^−1^ day^−1^)	RCR sorghum (mg mg^−1^ day^−1^)	*t* (p)
Maize‐rice (19)	3.83 ± 0.46	2.02 ± 0.25	–	3.78 (0.001)*
Maize‐sorghum (20)	2.74 ± 0.26	–	4.17 ± 0.47	–2.33 (0.031)*

^*^ RCR differs significantly in the two species tested in each two‐choice assay (paired Student's t test, *P* < 0.05).

#### 3.2.2 *No‐choice assays*

The RCR of leaf disks was significantly higher in maize compared with sorghum and rice. However, the RGR of larvae fed on sorghum was higher than in those fed on maize. This indicates that larvae convert the ingested sorghum to biomass more efficiently than in the case of maize, resulting in a significantly higher ECI in sorghum (51.6 ± 8.3%) in comparison with maize (18.7 ± 1.0%). On the other hand, both RGR and ECI were higher in maize in comparison with rice (Table 3).

**Table 3:** Nutritional indices (mean ± SE) of *S. nonagrioides* in no‐choice assays on the three cultivated hosts

Host (*n*)	RCR^a^ (mg mg^−1^ day^−1^)	RGR^b^ (mg mg^−1^ day^−1^)	ECI^c^ (%)
Maize (17)	4.85 ± 0.37	0.88 ± 0.06	18.7 ± 1.0
Sorghum (16)	2.87 ± 0.36*	1.19 ± 0.08*	51.6 ± 8.3*
Rice (8)	2.98 ± 0.40*	0.13 ± 0.02*	4.7 ± 0.8*
*F* (p)	10.4 (<0.001)	46.0 (<0.001)	94.6 (<0.001)

^a^ Relative consumption rate.

^b^ Relative growth rate.

^c^ Efficiency of conversion index.

* Significant differences with regards to the values recorded on maize (one‐way ANOVA followed by Dunnett's or T3 tests).

### 3.3 Oviposition preference on cultivated hosts

Females of *S. nonagrioides* showed preference for ovipositing on maize plants in comparison with sorghum or rice, so that the average fecundity on maize was significantly higher than that recorded in sorghum or rice (15‐ and 14‐fold, respectively). However, no significant differences were observed between hosts regarding fertility in maize‐sorghum or maize‐rice assays (Table 4). The average adult recovery rate per replicate was 80 ± 7% in maize‐sorghum assays and 93 ± 3% in maize‐rice assays.

**Table 4:** Fecundity and fertility (mean ± SE) of *S. nonagrioides* in choice assays between maize‐sorghum and maize‐rice

Host pair	n	Fecundity	*t* (p)	Fertility (%)	*t* (p)
Maize	18	387 ± 43	6.90 (<0.001)*	96.4 ± 1.1	−0.85 (0.486)
Sorghum		25 ± 16		99.4 ± 0.4	

Maize	15	449 ± 50	7.35 (<0.001)*	88.7 ± 6.8	0.45 (0.671)
Rice		32 ± 15		96.2 ± 1.6	

^*^ Significant differences from values obtained in maize (Student's *t*‐test, *P* < 0.05).

### 3.4 Free amino acid and free sugar content of the cultivated hosts

Free sugar content was significantly higher in rice leaf tissue compared to maize, whereas no differences were observed between maize and sorghum. On the other hand, the total free amino acid content was significantly higher in both sorghum and rice plants with regard to maize (Table 5).

**Table 5:** Percentage of free sugars and free amino acid content (mean ± SE) of maize, sorghum and rice leaf tissue

Host	Free sugars (%)	Free amino acids (%)
Maize	3.63 ± 0.32	0.256 ± 0.008
Sorghum	3.86 ± 0.30	0.631 ± 0.030 *
Rice	5.14 ± 0.28 *	0.475 ± 0.001 *
*F* (p)	6.86 (0.028)	90.28 (<0.001)

^*^ Significant differences from values obtained in maize (one‐way ANOVA followed by Dunnett's *t*‐test, *P* < 0.05).

## 4 DISCUSSION

*Sesamia nonagrioides* was able to lay eggs on all seven plants studied under no‐choice conditions. However, based on the results of the bioassays in which larvae were reared either on pieces of the plants or on whole plants, its larval cycle could only be completed in the three cultivated species tested – maize, sorghum and rice – as well as on the weed Johnsongrass, which shares similarities with sorghum, given that it is a hybrid of *S. bicolor* and *S. propinquum*.^44^ Common reed, giant reed and cattail were low‐quality hosts for larval development. Even though *S. nonagrioides* has been reported to feed on these species, all the studies reporting this behavior comprised African and Macaronesic populations,^45^, ^46^, ^47^, ^48^ so the differences observed between them and the Spanish one might be due to host specialization in *S. nonagrioides* populations from different areas in which host availability differs.

Different studies in noctuids have reported the important role of sensory and physical cues, such as surface texture or stem thickness, in the acceptability of a host species or plant part for oviposition.^49^, ^50^ In this vein, two other noctuid pests of maize, *Busseola fusca* and *Mythimna unipuncta*, have been observed to lay eggs on man‐made structures that resemble the narrow slit used for ovipositor insertion in maize plants, emphasizing the important role of this kind of cues in eliciting oviposition in both species.^51^, ^52^ Similarly, under laboratory conditions females of *S. nonagrioides* have been observed to lay viable eggs in artificial structures that mimic the tight gap between the stem and the leaf sheath (personal observation), which is used by the females of this species to insert the ovipositor.^53^ This suggests that, in the absence of its main host, *S. nonagrioides* females will lay eggs on a wide range of plant species, even when they are unsuitable for larval development, as already observed in this and other noctuid species.^54^, ^55^, ^56^ Our results agree with the narrower larval feeding range often observed in lepidopteran species in comparison with wider host acceptance for oviposition.^57^

When females could choose between the primary cultivated host (maize) and rice or sorghum in two‐choice bioassays, they showed a strong oviposition preference for maize, as evidenced by the more than 10 times higher fecundity recorded in this host with regard to the other two species. Even though sorghum and maize plants are phylogenetically very close and they share remarkable similarities in their architecture,^58^ the preference of *S. nonagrioides* females for laying eggs in maize rather than sorghum has been previously reported by Dimotsiou *et al*.^56^ This is in line with the better performance of *S. nonagrioides* observed on maize, since larvae generally showed higher mortality, delayed developmental time, reduced growth, smaller larval and pupal size, shorter adult life span and reduced fecundity and fertility in the alternative hosts in comparison with maize in both larval performance assays. These results are consistent with the ‘preference‐performance’ hypothesis that has been observed in several lepidopteran species,^59^, ^60^ which predicts that, generally, adults lay their eggs preferentially in hosts that are optimal for the development of their offspring.^61^ The differential development observed depending on the host could lead to asynchronies in the biological cycles, which in turn would result in different times of emergence of the adults from different plant species in the field. This could have important implications in terms of resistance management, as the main function of the refuges is to provide susceptible adults that will emerge at the same time as the resistant adults that may emerge in Bt maize fields. Therefore, developmental delay together with poorer quality adults produced in alternative hosts would make them not a good option as refuges for use within the HDR strategy.

Sugars and amino acids detected by chemoreceptors have been reported to play a major role in discrimination between plants for oviposition in some lepidopteran species.^21^, ^62^, ^63^ However, results are inconclusive when it comes to noctuid species.^22^, ^64^ Our study does not reveal an oviposition preference of *S. nonagrioides* for any of the plants based on their free sugars content, since, even though no differences in this parameter were found between maize and sorghum leaves, adults laid significantly more eggs on maize. However, maize had a significantly lower value in the total content of free amino acids in comparison with sorghum and rice, which could partially explain the differential oviposition preference between maize and these species.

The preference for laying eggs on maize shown by *S. nonagrioides* did not match the feeding preference for sorghum compared to maize shown by second instar larvae in two‐choice bioassays, as expressed by RCR values. This contradiction has also been observed in *S. exigua*, which preferred maize for oviposition and other plant species for larval feeding,^65^ but it differs from results reported in other species, where female oviposition preference for different host plants was positively correlated with larval feeding preference.^21^, ^66^, ^67^ A significantly higher content of free amino acids, which have been proved to stimulate feeding in other noctuid pests,^38^, ^68^ was detected in sorghum leaf tissue compared to maize. However, this would not be a major driver of *S. nonagrioides* larval preference and performance, since, even though rice contained higher levels of free amino acids than maize, larvae consumed more maize in maize‐rice choice assays and showed higher rates of relative consumption, growth and efficiency of conversion in maize than in rice under no‐choice conditions. In the same line, the results of choice and non‐choice assays show that the higher content of free sugars recorded in rice leaves in comparison with maize or sorghum did not stimulate feeding in *S. nonagrioides*, in contrast to the phagostimulant effect of sugars reported in other insect species.^39^, ^69^

On the contrary, the low relative growth and efficiency of conversion rates observed in larvae fed with rice leaf disks suggest a low nutritional quality and digestibility of this tissue for *S. nonagrioides*. This might not prove applicable to rice stems, given that a small percentage of *S. nonagrioides* larvae that were placed as neonates in rice plants completed the larval cycle. Nevertheless, even though a high proportion of larvae was recovered from infested rice plants, these larvae were developmentally delayed and their growth had been restricted in comparison with larvae recovered from maize plants.

Altogether, our results indicate that only three out of the six tested species were suitable hosts for *S. nonagrioides*, apart from maize. Nevertheless, none of the potential alternative hosts tested here should be used as natural unstructured refuges for Bt maize and considered within the HDR strategy. This is because even if these plant species are present and abundant near or within (i.e. Johnsongrass) Bt maize fields in Spain and they coincide with maize in both space and time, they do not comply with other requirements that must be met by unstructured refuges, that is, that they are able to host a large population of high‐quality moths, and that no asynchrony exists between these moths and those emerging from Bt fields.^70^, ^71^, ^72^ For some polyphagous target pests, natural refuges can be very effective, if the host plants in the refuge can produce sufficient numbers of high‐quality moths.^71^ Thus, natural hosts have been proved to contribute to delay resistance development to Bt cotton in China and the USA in the polyphagous and highly dispersive pests *Helicoverpa armigera*, *H. zea* and *Heliothis virescens*.^18^, ^19^, ^73^ However, they have not been considered as effective refuges for Bt maize for *Ostrinia nubilalis* in France and the USA, and for several stem‐boring pests of maize in Africa.^72^, ^74^, ^75^ Based on our findings, the tested hosts would also not be suitable for *S. nonagrioides*, which in Spain is considered an oligophagous or even facultatively monophagous species on *Z. mays*. Nonetheless, it must be kept in mind that *S. nonagriodes* may use these plants as refuges for short periods of time under adverse circumstances, e.g. to escape from Bt maize.^76^

The results reported in this study confirm the premises assumed in the resistance evolution model of *S. nonagrioides* to Bt maize in the Ebro Valley regarding the low number of wild or cultivated alternative hosts plants for this noctuid pest.^4^ Furthermore, they suggest that unstructured refuges composed of the tested plant species would not help delay the development of resistance to Bt maize. Therefore, refuges for susceptible individuals should continue to be composed of non‐Bt maize plants, and it is desirable that compliance with refuge requirements increases from the 92% reported in 2017^77^ in maize‐based agro‐ecosystems.
